# The bidirectional relationship between CFTR and lipids

**DOI:** 10.1038/s42003-020-0909-1

**Published:** 2020-04-20

**Authors:** Kirsten A. Cottrill, Carlos M. Farinha, Nael A. McCarty

**Affiliations:** 1 Molecular and Systems Pharmacology PhD Program, Emory University, Atlanta, GA USA; 2 Biosystems and Integrative Sciences Institute, Faculty of Sciences, University of Lisboa, Campo Grande, 1749-016 Lisboa, Portugal; 3 Department of Pediatrics and Children’s Healthcare of Atlanta, Center for Cystic Fibrosis and Airways Disease Research, Emory University School of Medicine, Atlanta, GA USA

**Keywords:** Biochemistry, Biophysics, Cell biology, Physiology

## Abstract

Cystic Fibrosis (CF) is the most common life-shortening genetic disease among Caucasians, resulting from mutations in the gene encoding the Cystic Fibrosis Transmembrane conductance Regulator (CFTR). While work to understand this protein has resulted in new treatment strategies, it is important to emphasize that CFTR exists within a complex lipid bilayer — a concept largely overlooked when performing structural and functional studies. In this review we discuss cellular lipid imbalances in CF, mechanisms by which lipids affect membrane protein activity, and the specific impact of detergents and lipids on CFTR function.

## Abstract (web summary)

In this review, Cottrill et al. discuss how the Cystic Fibrosis Transmembrane conductance Regulator (CFTR) interacts with the membrane into which it is inserted. They summarize recent insight into the ways lipids are imbalanced in CF epithelia and how the lipid environment affects CFTR.

## Introduction

**Fig. 1:**
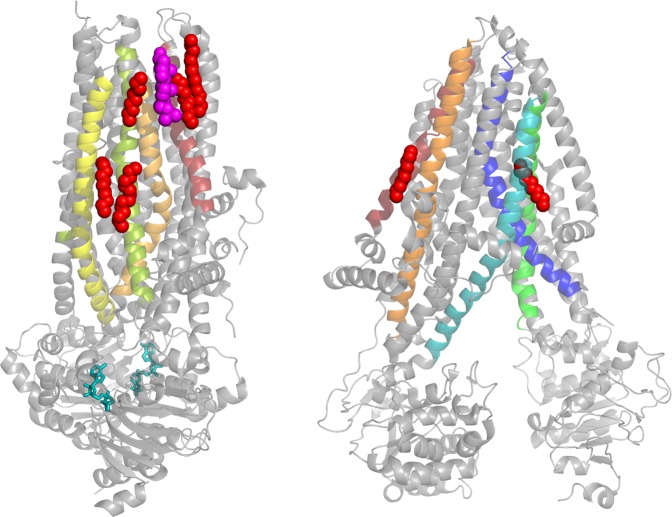
(Left) A representation of phosphorylated, ATP-bound human CFTR (PDB: 6MSM), with transmembrane helix 1 (TM1) in red, TM3 in orange, TM4 in yellow, and TM6 in yellow–green. The ATP molecules are teal, cholesterol is magenta, and other hydrocarbon chains are raspberry. *(*Right) A representation of dephosphorylated, apo-ATP zebrafish CFTR (PDB: 5UAR), with TM1 in red, TM2 in dark orange, TM9 in green, TM10 in teal, and TM 12 in blue. The hydrocarbon chains are raspberry.

Cystic Fibrosis (CF) affects more than 70,000 individuals worldwide. Currently, lung failure is the leading cause of death, although many patients suffer from pancreatic insufficiency, wherein digestive enzymes are not delivered from the pancreas to the gastrointestinal tract^1^. In 1989, the gene and protein associated with CF were identified and named the Cystic Fibrosis Transmembrane conductance Regulator (CFTR)^2,3^. CFTR is a member of the ATP-Binding Cassette Transporter superfamily (ABCC7). However, whereas all other ABC transporters use ATP to power an enzymatic function, CFTR is the only ABC transporter that functions primarily as an ion channel. Specifically, CFTR conducts chloride and bicarbonate. CFTR’s domain architecture includes two transmembrane domains (each with six transmembrane helices (TMs)), two nucleotide binding domains, and a regulatory R-region (Fig. 1). Opening of the CFTR channel requires phosphorylation of the R-region by protein kinase A (PKA) and binding and subsequent hydrolysis of ATP at the nucleotide binding domains^4^. Interestingly, the entirety of CFTR is sensitive to mutation, with some 2000 genetic variants having been described. However, F508del-CFTR is by far the most common variant, with a ~70% allele frequency in U.S. patients (https://www.cftr2.org/). Even the ultimate effect of these mutations on CFTR is complex, which is why seven different classes of mutations have been described (Table 1)^5,6^.

**Table 1:** The seven classes of CFTR defects, with examples.

Class	Explanation	Example
I	defective synthesis of the protein	G542X
II	defective trafficking to the cell membrane	F508del
III	decreased ability to open, even though it is present at the cell surface	G551D
IV	decreased ability to conduct ions, even though the gate can open	R347P
V	decreased amount of post-translationally processed, fully active CFTR	A455E
VI	decreased stability at the surface	N287Y
VII	absence of mRNA	del2,3(21kb)

For a subset of these mutations, small-molecule therapeutics exist that increase the activity of CFTR. The first FDA-approved drug, VX-770 (Ivacaftor, KALYDECO^®^)^7^, is a gating potentiator that helps certain CFTR mutants conduct more chloride upon activation by sub-maximal phosphorylation^8^. The second drug, VX-809 (Lumacaftor), is a trafficking corrector that helps certain CFTR mutants reach the cell membrane^9^. Lumacaftor is only ever administered in combination with Ivacaftor, prescribed as the combination drug ORKAMBI^®^. Unfortunately, Ivacaftor decreases the efficacy of Lumacaftor in patients with F508del-CFTR^10,11^. To improve efficacy and to minimize side effects, another corrector, VX-661 (Tezacaftor), was developed and has been effective when used with Ivacaftor (combination therapy SYMDEKO^®^ in the U.S. or Symkevi in the E.U.)^12^. Most recently, another corrector, VX-445 (Elexacaftor), was approved in combination with Ivacaftor and Tezacaftor (triple combination therapy TRIKAFTA^TM^ in the U.S.)^13^. The exact mechanisms by which these drugs work are still unknown, although this knowledge is imperative for refining the next class of drugs toward greater efficacy.

In addition, to develop more effective drugs, we must understand CFTR in a more complete manner. However, the interaction between CFTR and the membrane it resides in has not been extensively explored, though there are known lipid imbalances in CF patients. This gap in knowledge needs to be addressed, as lipids are known to affect the function and stability of other ABC transporters and ion channels. It is, therefore, very likely that lipids modulate the function and stability of CFTR. This review seeks to explore the potential avenues by which CFTR and lipids interact by reviewing the current knowledge about the ways by which loss of functional CFTR affects lipids, and the ways in which lipids affect CFTR. We also discuss lipid interactions of other ion channels and ABC transporters, as these may inform us on lipid interactions relevant to CFTR function.

## Lipid imbalances in CF epithelia

Dyslipidemia has been reported in CF, especially in relation to pancreatic insufficiency and CF-related diabetes. These data are extensive and frequently conflicting, and have been reviewed elsewhere^14^. Here we focus mainly on lipid imbalances in CF airway and airway epithelial cells in human and mouse models in an effort to understand the roles lipids may play in the lung disease of CF patients and the lipid environment that CFTR experiences in these cells.

### Fatty acids are imbalanced in CF epithelia

Linoleic acid and arachidonic acid are omega-6 fatty acids. Linoleic acid is an essential fatty acid, meaning it has to be absorbed through the diet. Arachidonic acid can be absorbed through the diet or catabolized from linoleic acid. Docosahexaenoic acid is an omega-3 fatty acid that can be absorbed through the diet or produced from metabolism of eicosapentaenoic acid or α-linoleic acid. Whereas each of these fatty acids play a vital physiological role, imbalances between them can affect health, with an increased ratio of omega-6/omega-3 fatty acids inducing a pro-inflammatory state^15^.

Arachidonic acid was increased and docosahexaenoic acid was reported as decreased in nasal epithelial cells collected from people with CF, compared with control. Importantly, this imbalance was seen regardless of pancreatic sufficiency status, indicating that the modified high-calorie/high-fat diet of pancreatic insufficient people with CF was not the cause of this lipid imbalance^16^. In support of this, experiments were performed in either the commonly-used 16HBE bronchial epithelial cell line expressing WT-CFTR or with CFTR transiently knocked down (CF), or the IB3-1 CF cell line (bearing F508del/W1282X genotype) alongside IB3-1 cells virally transduced to express WT-CFTR (C38 cells). It is worth noting that cell lines are an imperfect model due to their genetic instability, and that patient-derived cells are considered the gold standard. Furthermore, the 16HBE pair is not a perfect model, as most CF patients express the trafficking mutant F508del-CFTR, which adds complexity not captured by using a cell line that produces no CFTR since the misfolded F508del-CFTR may induce an unfolded protein response. Conversely, the C38 control cells express both two mutated proteins and the WT protein, which is not representative of non-CF cells. Still, whereas both sets of model cell lines have limitations they indicate increased arachidonic acid concentrations alongside decreased linoleic and docosahexaenoic acid levels as compared with the control^17,18^.

Despite this overall decreased presence of linoleic and docosahexaenoic acid (DHA), CF cells showed an increased uptake of these lipids. Further investigation determined that there was a greater metabolism of linoleic acid into arachidonic acid via Δ5- and Δ6-desaturase, explaining the decreased abundance of linoleic acid. This increase in Δ5- and Δ6-desaturase activity could be due to a loss of proper CFTR function, which leads to an increase in intracellular calcium that activates calcium/calmodulin-dependent protein kinase kinase β (CaMKKβ), which activates 5’ AMP-activated protein kinase (AMPK). Inhibiting CaMKKβ or AMPK returns Δ5- and Δ6-desaturase to control levels^19^. The decreased levels of DHA are likely due to increased retroconversion of DHA to its precursor molecule^20^.

Overall, it seems that CF epithelial cells have reduced levels of docosahexaenoic acid and increased levels of arachidonic acid, leading to an imbalanced ratio of the two that indicates a pro-inflammatory state. The overall metabolic profile in these cells was reversed in CF cells, but not in non-CF cells, by supplementation of docosahexaenoic acid, which is known to downregulate expression of the desaturases^17,18,20^. While some clinical trials investigated supplementing DHA into the diets of people living with CF, there is more work to be done to determine the efficacy of this treatment and to determine if current FDA-approved therapies correct the fatty acid imbalance in CF epithelial cells^20^.

### Cholesterol is increased in CF epithelia

Cholesterol, the precursor for many vitamins and hormones, constitutes a large percentage of the lipid mass of the plasma membrane, has a large influence on the fluidity of the membrane, and clusters with sphingomyelin to form lipid rafts^21^.

HeLa cells transfected with constructs encoding F508del-CFTR and stained with filipin showed an accumulation of cholesterol^22^. Similarly, baby hamster kidney and Chinese hamster ovary cells transfected with constructs encoding F508del- or C1410X-CFTR mutants (usually trafficked from the ER to the lysosome, but that can traffic to the Golgi and cell surface when rescued by temperature shift to 27 °C) indicated an increase in free cholesterol as well as a redistribution of cholesterol from the Golgi to non-Golgi vesicular structures (thought to be late endosomes). This redistribution was intensified by temperature shift rescue of CFTR trafficking. Transfection with constructs encoding WT-, G551D- (gating defect mutation, no trafficking defect), and D572N-CFTR (trafficking defect mutant, not rescued by temperature shift) did not affect cellular cholesterol levels^23^. This suggests that when mutant CFTR escapes the ER quality control mechanisms and traffics to the surface, the mutants that are quickly targeted for degradation (e.g., F508del- and C1410X-CFTR) are trafficked through the endosomal pathway to the lysosome. This may disturb the ability of the endosomal pathway to process cholesterol, leading to cholesterol accumulation in endosomal structures. While this theory seems plausible, the fact that these studies were conducted in cells that do not normally express CFTR raises questions as to its validity in relevant cells.

More physiologically relevant data indicate that cholesterol accumulates intracellularly and in the cell membrane of CF airway cell lines and in the pulmonary epithelia of CFTR knockout mice fed a liquid diet, similarly to observations in Niemann-Pick Disease Type C cells^24,25^. This cholesterol accumulation seemed to cause some of the inflammatory imbalances common in CF, such as decreased nitric oxide synthase (NOS2) activity and decreased activity of signal transducer and activator of transcription-1 (STAT1), as correction of cholesterol concentrations by treatment with an HMG-CoA reductase inhibitor resolved these imbalances^25,26^.

The mouse data may be confounded by results showing that the liquid diet Peptamen causes a 300% increase of cholesterol in the lungs of mice with either residual CFTR activity or intestinal CFTR expression^27^. However, within the cell lines, the non-CF and CF counterparts are maintained in the same nutritional media conditions, yet these cell line pairs still show similarly increased cholesterol levels; this suggests that the cholesterol imbalance and its resulting inflammatory imbalance are likely caused by the primary CFTR defect and not by the diet. Still, little work has been done to quantify the relative levels of cholesterol in lung epithelial cells from people with CF, which is vital to confirm the relevance of this observed imbalance. A thin-layer chromatography analysis of tracheobronchial sections revealed that CF lung sections showed increased cholesterol content compared with non-CF lungs^28^. However, CF patients are encouraged to eat a high-calorie, high-fat diet, which is why it is difficult to distinguish in actual patient samples a primary defect from an acquired imbalance. Thus, though data from cell lines and mouse tissue as well as some patient data indicate that cholesterol is increased in airway epithelia, more work is necessary to confirm that this imbalance is due to the primary CFTR defect, to confirm that the mechanism established in non-epithelial cell lines is consistent with the mechanism in cells collected from people living with CF, and to understand the pathology of how this imbalance affects the wellbeing of people living with CF.

### Ceramides are imbalanced in CF epithelia

Ceramide is a sphingolipid implicated in pro-inflammatory and pro-apoptotic signaling, but also in proliferative signaling. Which signaling pathways are activated depends upon the acyl chain length^29^. Ceramide concentrations in cells are controlled by the direct synthesis of ceramide, production by the salvage pathway, and breakdown of sphingomyelin by sphingomyelinases (SMases) into ceramide and phosphocholine^30^. This balance between ceramide and sphingomyelin is important, as sphingomyelin and cholesterol cluster to form lipid rafts whereas ceramide clusters with itself to form larger lipid platforms that exclude cholesterol^21,31^. Many different proteins localize preferentially into these sphingomyelin rafts or ceramide platforms, bringing them into close proximity to each other to increase the efficiency of their interactions^31^. Generally, these protein clusters involve various partners of a signaling mechanism, increasing the efficiency of their influence on their specific signaling pathways. Importantly, CFTR clusters into both sphingolipid rafts and ceramide platforms; the impact of this on channel mobility and activity will be discussed below^32–34^.

The activity of CFTR in these different microdomains is relevant to CF, as ceramide levels are imbalanced in CF. Originally, evidence indicated that ceramide levels were increased in lung epithelia from CF patients^27^, and in epithelial cell lines expressing mutant CFTR^35^. However, the ceramide antibody used in this study was found to non-specifically bind many other types of lipids, leading to doubt about the validity of these results^36^. Alternatively to this model, and utilizing much more accurate mass spectrometry methods, some mouse studies using a whole-animal CFTR knockout strain revealed decreased ceramide content in the total lung homogenate, but an increase in the ratio of long chain ceramides to very long chain ceramides as compared with control mice^37–39^. Importantly, this imbalance was found regardless of whether the mice were fed the liquid Peptamen diet or the standard chow diet like the non-CF control mice, indicating that altered diet was not the cause of ceramide imbalance. This ceramide imbalance could contribute to the chronic inflammation seen in CF patient lungs as long-chain ceramides are pro-inflammatory while very-long-chain ceramides are anti-inflammatory (see Ghidoni et al.^40^, for details on ceramide’s involvement in the pathophysiology of CF and other inflammatory lung diseases). Importantly, total lung homogenate includes much more than just epithelial cells, so these data must be considered carefully since it is unclear what cell types are contributing to this imbalance.

To focus on epithelial cells, studies comparing CFBE41o- cells (a bronchial epithelial cell line homozygous for the F508del mutation but not expressing any CFTR protein, representing the CF phenotype), and CFBE41o- cells transduced with lentiviral vector containing WT-CFTR (representing the non-CF phenotype) indicated a ceramide imbalance similar to that described above for CF mice^38^. However, as mentioned previously, these cells do not represent the trafficking defect of the most common CFTR mutation, which is important to consider since this trafficking mutation could worsen the imbalance seen following loss of CFTR. Further studies using primary epithelial cells from individuals with CF are necessary to more fully understand the ceramide imbalance in a clinical setting.

Two drugs are currently in clinical trials targeting the ceramide imbalance. Based on the original data showing that ceramide is increased in CF, amitriptyline is being explored as a functional acid-SMase inhibitor that decreases ceramide levels. Interestingly, amitriptyline increased the forced expiratory volume in the first second (FEV_1_) and body mass index in people with CF^41,42^. However, amitriptyline is a tricyclic antidepressant with many effects. Furthermore, amitriptyline inhibits acid-SMase by inducing its dissociation from the lysosomal membrane, causing its degradation^43^. This very nonspecific mechanism likely affects other lysosomal membrane-associated proteins. Based on the more widely accepted data showing a ceramide imbalance in CF, fenretinide, a retinoic acid derivative, also is being explored. In Phase I trials, fenretinide restored the ceramide imbalance in blood samples^38^. In mice, it was found to decrease the bacterial burden of CF mice acutely infected with *Pseudomonas aeruginosa*^37^. In CFBE41o- cells, fenretinide re-balances ceramide levels by decreasing ceramide synthase 5 activity while maintaining ceramide synthase 2 activity. However, the study does not report any differential expression of ceramide synthases in CF cells compared with controls, leaving the underlying mechanism still unclear. Understanding the mechanism by which loss of CFTR function leads to an imbalance in ceramide species, how this imbalance affects the disease state, and how to correct the imbalance, are important future steps toward developing new alternative treatments for CF.

### Gangliosides are imbalanced in CF epithelia

Gangliosides are a type of sphingolipid comprised of a ceramide base and an oligosaccharide headgroup. Importantly, *P. aeruginosa* binds to asialo-monosialotetrahexosylganglioside (asialo-GM1)^44^. Asialo-GM1 is increased in the 9HTEo^-^ tracheal epithelial cell line overexpressing the R-region (functionally inhibiting CFTR, mimicking but not replicating CF), as determined by an anti-asialo-GM1 antibody^45^. This could explain the increased adherence of *P. aeruginosa* to CF epithelial cells. Potentially complementary data come from Calu-3 bronchial epithelial cells treated with short hairpin RNA against CFTR (functional knockout, not accounting for defective protein folding or trafficking), which show reduced GM1 levels as compared with the control Calu-3 cells, as detected by thin layer chromatography and cholera toxin B staining^46^. It is possible that the GM1 levels are reduced because there is an increased conversion into asialo-GM1, but experiments have not been conducted to determine the mechanism of either the decrease in GM1 or the increase in asialo-GM1. Importantly, though, this reduction of GM1 seemed to cause delayed wound repair in the Calu-3 cells. Restoration of GM1 into the cells recovered this delay^46^. More work is necessary to better understand the imbalance of these lipids, the mechanism by which loss of CFTR function leads to the imbalance, and the clinical relevance of this imbalance.

### Sphingosine and sphingosine-1-phosphate are imbalanced in CF epithelia

Ceramide is degraded by acid ceramidase (acid-CDase) into sphingosine. Based on anti-sphingosine antibody staining, sphingosine is decreased in nasal epithelial cells from CF patients and in CF mouse airway epithelial cells. More reliable mass spectrometry found similarly increased sphingosine levels in tracheal epithelial cells from two different CF mouse models, though these experiments need to be repeated in human samples^47,48^. The mechanism for this decreased sphingosine mass has not been determined. Importantly, though, rescue of sphingosine levels by inhalation of acid-CDase or sphingosine itself prevented pulmonary *P. aeruginosa* infection in CFTR knockout mice, indicating that it is an important molecule for antibacterial resistance^47^.

Sphingosine is phosphorylated by sphingosine kinases into sphingosine-1-phosphate (S1P), a signaling lipid generally associated with proliferation and antagonization of apoptosis^49^. S1P initiates many different signaling cascades via extracellular activation of its five GPCR receptors (S1PR_1_ -S1PR_5_), with S1PR_3_ being the most highly expressed receptor in human bronchial epithelial cells^49,50^. Importantly, CFTR is thought to import S1P, sequestering it from its GPCRs. CFTR transports S1P into the cell independently of its chloride channel function in C127 mouse mammary epithelial cells^51^. Unfortunately, this study did not test the S1P transport of a gating mutant such as G551D-CFTR, but rather focused on the trafficking mutant F508del-CFTR. If S1P transport occurs independently of the chloride channel activity, it is important to know if S1P transport is altered in CFTR gating mutants as well, and whether this function is sensitive to the known small-molecule CFTR modulators described above. Furthermore, it was not determined whether CFTR directly transports S1P or if it facilitates S1P transport via another mechanism. Overall, because CFTR affects S1P trafficking, sphingosine levels appear decreased in CF airway epithelia, and because this decreased sphingosine is associated with increased bacterial infection, it is vital to understand the mechanisms by which CFTR dysfunction leads to imbalanced sphingosine levels in order to contextualize the disease state of CF more accurately. Further, it is important to understand if current modulator therapies rebalance these lipids and reduce the susceptibility to bacterial infection, which is a major concern for people living with CF.

## How lipids generally affect membrane protein activity

**Fig. 2:**
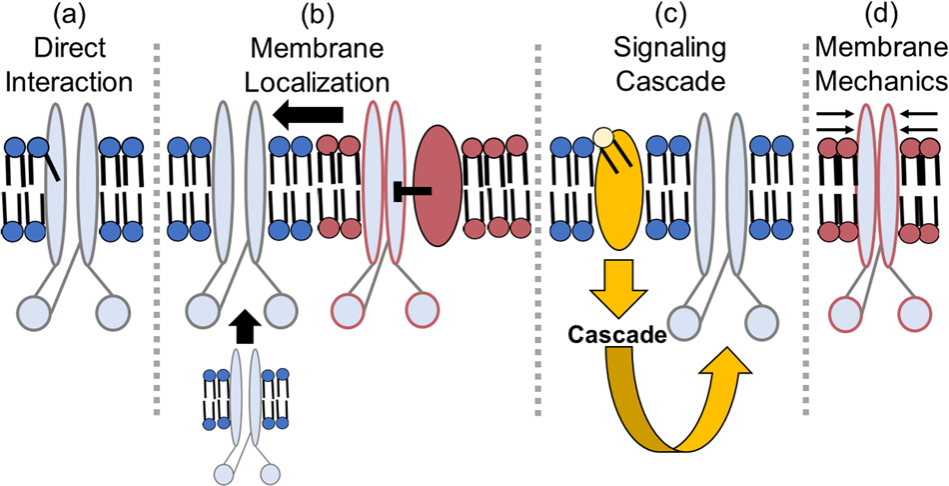
A graphical representation of the various ways that lipids can affect membrane protein activity. In this case, the representative protein (light blue) is drawn to resemble the structure of the CFTR ion channel. A red outline indicates that the protein is inhibited. **a** Lipids can have a direct, physical interaction with the protein, as shown by the tail protruding into the protein. **b** Lipids can affect the membrane localization of the effector protein, either affecting its trafficking to the plasma membrane or localizing it to domains with other proteins that may affect its function. **c** Lipids can initiate a signaling cascade that results in post-translational modifications to the protein that affect its function. **d** Lipids, based on their flexibility and curvature, can impose mechanical forces on the protein that affect its ability to move and function.

It is important to discuss well-characterized interactions between lipids and membrane proteins, which could hint at possible lipid-CFTR interactions that have yet to be characterized. There are four main ways by which lipids can affect ion channels: By direct allosteric interactions, by changing their surface localization, by changing signaling cascades that modify the protein, and through alterations of membrane mechanics such as fluidity (Fig. 2). Frequently, these mechanisms overlap, making it hard to define the system fully. Still, we will attempt to explore each individually using representative examples that illustrate the concept (Box 1).

Box 1 How lipids interact with ABC transporters Since CFTR is an ABC transporter, knowledge about the lipid interaction of the larger ABC transporter family may be useful to understanding CFTR. ABC transporters from many different subfamilies transport lipids (see below)^83, 86^. Aside from serving as transported substrates, lipids can have a large impact on ABC transporter stability and function^87^. For example, some uncharged phospholipids had stimulatory effects on ABCB1 and its ATPase activity whereas negatively charged phospholipids were inhibitory^88^. Interestingly, this study also determined phospholipids that co-purified with ABCB1, indicating tight binding. Another study showed that depletion of cholesterol resulted in a significant decrease in basal ATPase activity of ABCB1 whereas repletion of cholesterol recovered ATPase activity^89^. Further work on purified human ABCB1 reconstituted into liposomes with varying concentrations of cholesterol confirmed that cholesterol increased basal ATPase activity and that cholesterol co-purified with ABCB1^90^. Thus, these data indicate that ABCB1 is tightly bound to many lipids, and that these lipids affect the activity of this protein.

ABCB1 is not the only ABC transporter affected by lipids, and not all ABC transporters are affected in the same way by the same lipids. The exact mechanism by which these lipids affect the activity of ABC transporters is not fully understood. Structural data will be needed to confirm possible direct interactions. Currently, only a few structures of human ABC transporters other than CFTR have been solved at high resolution. Of the solved structures, ABCG2 (PDB: 6HIJ)^91^ and ABCB10 (PDB: 3ZDQ, 4AYT, 4AYX, 4AYW) show bound lipids^92^. More work is required to improve methods of purifying and imaging other ABC transporters with potential lipid interactors. Since CFTR is an ABC transporter, the refinement of these methods will inevitably facilitate a better understanding of CFTR as well, which is important for being able to understand CFTR in its endogenous lipid environment.

**A list of some ABC Transporters with lipid substrates, along with their associated diseases**

**Table:** 

**Name**	**Substrate(s)**	**Disease**
ABCA1	cholesterol and phospholipids	Tangier
ABCA4	N-retinylidene-phosphatidylethanolamine	Stargardt
ABCA7	phosphocholine and sphingomyelin	Alzheimer’s
ABCB1	glucosylceramides and phospholipids	Inflammatory Bowel Disease
ABCB4	phosphatidylcholine	Progressive Familial Intrahepatic Cholestasis 3
ABCC1	glucosylceramide, sphingomyelin, and sphingosine-1-phosphate	
ABCC1–ABCC4	bile salts	ABCC2: Dubin-Johnson Syndrome
ABCD1–3	fatty acyl-CoA	ABCD1: Adrenoleukodystrophy
ABCG1, ABCG2	sterols

### Direct allosteric lipid interactions affect membrane protein activity

One interesting example of direct lipid interactions affecting ion channel activity involves the inwardly-rectifying potassium channel 2 (Kir2). These channels localize to cholesterol-rich microdomains^52^, although Kir2 channels are actually inhibited by cholesterol. Enriching cells with cholesterol by treating them with cholesterol-loaded methyl-β-cyclodextrin (MβCD) caused whole-cell Kir2 current to decrease. Inversely, depleting cells of cholesterol by treating them with empty MβCD caused whole-cell Kir2 currents to increase^52,53^. To determine if the inhibitory effects of cholesterol were specific (rather than caused by bulk membrane fluidics), cholesterol was exchanged with epicholesterol (a stereoisomer of cholesterol) by treating cells with epicholesterol-loaded MβCD. This replacement of cholesterol with epicholesterol caused an increase in whole-cell Kir2 currents, similarly to simply removing cholesterol, implicating a stereo-specific inhibitory interaction between cholesterol and Kir2 channels^53^. This inhibition of Kir2 by cholesterol was based on a direct binding of cholesterol to the channel^54^. This exemplifies one of many direct allosteric interactions that lipids can have with membrane proteins, how that interaction can affect a protein’s activity, and how experiments can be set up to determine the interaction.

### Lipid-mediated membrane surface localization affects membrane protein activity

Surface localization of a protein involves at least two major concepts: (1) the total amount of protein in the cell membrane overall, and (2) the relative distribution of the protein within specific microdomains of the cell membrane. Cholesterol, for example, impacts both of these properties for many membrane proteins. Regarding the first concept, cholesterol is required to maintain the surface expression of the epithelial sodium channel (ENaC). In mouse cortical collecting duct, loss of cholesterol caused an apparent decrease in basal ENaC activity due to a decrease in the number of channels present at the cell surface^55^. Regarding the second concept, cholesterol affects the localization of PDZ domain-containing proteins within the plasma membrane. A PDZ domain binds the PDZ binding-domain motif of another target protein. Generally, there are multiple PDZ domains within a single protein, allowing it to act as a scaffold to bring two proteins with PDZ binding-domain motifs into close proximity^56^. Frequently, these PDZ domains contain a cholesterol-binding sequence, and those that do bind cholesterol tend to depend upon this lipid to localize to the plasma membrane^57^. Thus, cholesterol is important for localizing some PDZ-interacting proteins. One important PDZ domain-containing scaffold protein to consider is the sodium-hydrogen antiporter 3 regulator 1 (NHERF1), which binds CFTR (among other proteins) and stabilizes its localization to cholesterol-rich microdomains^58,59^. Again, cholesterol serves just as an example of one way in which lipids can affect membrane protein localization, and how that membrane localization can affect the apparent activity of the protein. This is an important concept to keep in mind in studying the effects of lipids on CFTR.

### Lipid-mediated signaling cascades affect membrane protein activity

Aside from lipid rafts, which are enriched in sphingomyelin and cholesterol, ceramide platforms also have been described, which exclude cholesterol^31^. Localization to these domains can alter protein activity. For example, apoptosis antigen 1 (APO-1) is activated when localized to ceramide-rich platforms^60^. Activation of APO-1 initiates a signaling cascade ultimately leading to cell apoptosis. Ceramide and some of its derivatives activate many different signaling cascades. For example, in oligodendrocytes, ceramide inhibits Kir channels via Ras and protein kinase C (PKC), both of which interact with Raf-1^61^. As seen by this example, since lipids are able to affect membrane protein activity by activating signaling cascades, it is important to study the potential lipid imbalances in CF and how they can affect the signaling cascades involved in modulating CFTR activity.

### Lipid-dependent membrane mechanics affect membrane protein activity

Lipids also can affect membrane protein activity by altering characteristics of the membrane environment such as hydrophobic thickness, curvature, and flexibility. All membrane proteins have an optimal transmembrane-domain length. Bilayers that are too thick or too thin can shift the hydrophobic contacts of the protein, causing either the protein, the lipid bilayer, or both to deform to minimize exposure of hydrophobic surfaces to water^62,63^. Besides thickness, deformation can occur by pressure exerted by the curvature of the membrane as well. Deformation of the protein may cause a change in the activity of that protein, perhaps by promoting protein conformations that enhance or decrease function. Furthermore, ion channels are conformationally dynamic proteins; whenever a channel opens, it deforms the membrane around it to make room for a pore through the channel. These membrane deformations include stretching, compressing, or bending the bilayer or protein^63–65^. Deformation of the membrane or protein requires energy, and the amount of energy depends on the flexibility of the “annular lipids” immediately surrounding and interacting with the membrane protein. Thus, the immediate lipid environment of a transmembrane protein may affect protein activity based on the mechanical stress it encounters.

This is exemplified by the bilayer deformations associated with the conformational movements of the Ca^2+^-ATPase (SERCA1)^66^. X-ray crystallography studies of SERCA1 incubated with soy bean phosphatidylcholine and octaethyleneglycol mono-*n*-dodecyl ether (C12E8) detergent were integrated with solvent contrast modulation experiments to resolve the protein and its surrounding bilayer in four different reaction states (1: two Ca^2+^ associated, 2: two Ca^2+^ and ATP associated, 3: transition state where two Ca^2+^ and ADP and P_i_ are associated, and 4: phosphorylated protein stabilized with inhibitor thapsigargin). The resulting SERCA1 structures were then simulated in a 1,2-Dioleyol-sn-glycero-phosphocholine (DOPC) bilayer. Transitioning between State1 and State3, the protein tilts 18°. The tilting between each state causes distortion of the surrounding membrane. Thus, membrane flexibility is required in order to accommodate this massive movement of SERCA1 while maintaining hydrophobic contacts. Lack of flexibility would therefore in theory lead to an inhibition of SERCA1 function. This serves as an example of the overarching principle that membrane mechanics, dictated by the lipids comprising the membrane, affect membrane protein activity. This concept has not yet been applied to CFTR when studying its activity, but is of vital importance to gathering a more complete understanding of CFTR.

### Detergents and membrane lipids affect CFTR stability and ATPase activity

Whereas all ABC transporters are difficult to work with, CFTR is notoriously difficult to purify for biochemical and biophysical studies, in part because of its propensity to aggregate and its instability in solution. Nonetheless, some recent successes have led to important new structural insights for this channel.

Currently, the highest-resolution structures of CFTR have come from the Chen lab using single-particle cryo-electron microscopy (Table 2). All six of these CFTR structures were purified initially in 0.2–0.25% cholesteryl hemisuccinate and 1–1.25% 2,2-didecylpropane-1,3-bis-β-D-maltopyranoside (LMNG). It is important to note a critical downfall to these current CFTR structures – none of the outward-facing structures are actually open, as they do not have a conducting pore that spans the entire thickness of the membrane. This issue of these structures lacking a complete pore is compounded by the fact that the purified CFTR itself was never assessed for ion channel activity. It would make sense for the E1371Q-CFTR stabilized mutant to have incomplete channel activity, as this removes the ATP hydrolysis function, which is required to reach the full conducting state^67^. However, even the WT-CFTR that was used for the closed structures was not assessed for ion channel function or ATPase activity after purification. Thus, it is unknown whether the purification process denatured CFTR to any extent, and whether this explains the lack of a pore in the final structures.

**Table 2:** The current high-resolution structures of CFTR from the Chen lab.

Origin	Mutation	State	Resolution	Year	PDB ID
Human	–	Dephosphorylated, Apo-ATP, inward-facing	3.87 Å	2017	5UAK^84^
Human	E1371Q	Phosphorylated, ATP-bound, outward-facing	3.2 Å	2018	6MSM^68^
Human	E1371Q	Phosphorylated, ATP-bound, outward-facing, Ivacaftor-bound	3.3 Å	2019	6O2P^70^
Human	E1371Q	Phosphorylated, ATP-bound, outward-facing, GLPG1837-bound	3.2 Å	2019	6O1V^70^
Zebrafish	–	Dephosphorylated, Apo-ATP, inward-facing	3.73 Å	2016	5UAR^71^
Zebrafish	E1372Q	Phosphorylated, ATP-bound, outward-facing	3.37 Å	2017	5W81^85^

Interestingly, lipids appear in a few of the CFTR structures described above, either co-purified from the cell or introduced during purification. The phosphorylated, outward-facing human CFTR shows a cholesterol molecule bound toward the top of the TMD (Fig. 1a)^68^. Importantly, the more recently released phosphorylated, outward-facing human CFTR bound to GLPG1837 shows a cholesterol bound to the same spot. While CFTR is predicted to have eight cholesterol binding sites according to molecular docking simulations against the inward-facing zebrafish CFTR structure^69^, none of these sites match the site of bound cholesterol observed in the outward-facing human CFTR structure^68^. There are also five hydrocarbon chains of varying lengths bound to the outward-facing human CFTR structure that were also seen bound to the same pockets in the outward-facing human CFTR structure bound to the CFTR potentiator GLPG1837^70^. There were also six hydrocarbon chains associated with the outward-facing human CFTR structure bound to VX-770, with most of them in the same pockets as the other structures^70^. The hydrocarbons are likely the only resolved portions of larger phospholipids, which would have had to come from the original HEK plasma membrane since phospholipids were not added during purification. The inward-facing zebrafish CFTR structure also shows two hydrocarbon chains (Fig. 1b)^71^. Neither of these chains matches the binding location of the chains in the human structure discussed above. It should, however, be noted that the human structure is outward-facing, whereas the zebrafish structure is inward-facing. It is possible that the lipid interactions change based on the state of the channel, as noted above for the SERCA1 pump^66^. However, this remains uncertain as specific lipids were not seen in the inward-facing human or the outward-facing zebrafish CFTR structures. Future experiments should work toward understanding the effect(s) that these cholesterol molecules and acyl chains have on the structure and function of CFTR.

Further evidence of important lipid-CFTR interactions comes from the observation that adding a 4:1 (w/w) ratio of 1-palmitoyl-2-oleoyl-sn-glycero-3-phosphocholine (POPC) to 1-palmitoyl-2-oleoyl-sn-glycero-3-phosphoethanolamine (POPE) with anionic detergents helped stabilize NBD1 of CFTR^72^. Though POPC and POPE may stabilize NBD1, molecular docking simulations indicate that the NBDs preferentially bind the phosphoserine head group over PC or PE^73^. Furthermore, 1-palmitoyl-2-oleoyl-sn-glycero-3-phosphoserine (POPS) stabilized full-length CFTR more than POPC or POPE and conferred higher ATPase activity to CFTR than POPC or POPE. Interestingly, this study also demonstrated that brain PS, which has mostly C_18_ acyl chain lengths, helped CFTR maintain an eight-fold higher ATPase activity than any previously reported and a 10–12 °C higher thermal stability than detergent alone.

### Arachidonic acid affects CFTR activity

As mentioned above, arachidonic acid levels are increased in CF cells as compared with control. This is an important context to consider, as the effect of arachidonic acid on CFTR activity is complex and still incompletely understood. In detached membrane patches from Calu-3 pulmonary epithelial cells, intracellularly-applied arachidonic acid inhibited already activated CFTR^74,75^. Further studies suggested that arachidonic acid directly inhibited CFTR by inserting itself into the pore from the cytoplasmic face, interacting with the positively charged residues K95 and R303. Identification of these amino acids required mutating residues into an uncharged alanine, a polar glutamine, and a negatively charged glutamate, and then determining the K_d_ for arachidonic acid-mediated inhibition of these mutant CFTRs. However, there is no information regarding the effect of these mutations on single-channel CFTR activity in the absence of arachidonic acid, which is important to consider. For example, K95E, which induces the highest apparent K_d_ for arachidonic acid, may just stabilize the open state of CFTR rather than prevent arachidonic acid from blocking the pore. If arachidonic acid is not acting as a pore blocker but rather as an allosteric modulator, mutations stabilizing the open state would cause an apparent increase in K_d_.

To complicate the story further, arachidonic acid is not always inhibitory. In polarized Calu-3 monolayers, extracellularly-applied arachidonic acid slightly activated CFTR currents via a cyclooxygenase (COX) and/or arachidonate 5-lipogenase (5-LO) metabolite^76^. The mechanism by which activation occurs has not been determined. The validity and relevance of arachidonic acid’s positive and negative interactions with CFTR are vital to understand given that studies have found elevated arachidonic acid in CF cells (see above). Furthermore, it is important to determine if docosahexaenoic acid has the inverse effect on CFTR. Understanding these effects will help researchers determine the feedback loops present in CF that facilitate chronic inflammation, potentially uncovering new therapeutic targets that address multiple aspects of the disease and interrupt detrimental feedback loops or support helpful feedback loops.

### Ceramide and its derivatives affect CFTR activity

The effect of ceramide on CFTR activity is also complex and incompletely understood, in part because ceramide and its derivatives are involved in so many signaling cascades, depending in part on the fatty acid chain length, which also controls the effects on membrane mechanics. Whereas data discussed earlier indicate that there is a ceramide chain length imbalance in CF, unfortunately, research up to this point has not specifically accounted for ceramide chain length when evaluating the direct effects of ceramide on CFTR activity, and is something the field should consider moving forward.

In *Xenopus* oocytes, bacterial SMase inhibited WT-CFTR and, to a lesser degree, CFTR with the R-region deleted^77,78^. In studies from the McCarty lab, bath-applied recombinant SMase inhibited CFTR channels studied by cell-attached patch clamp recording where SMase was added to the bath and thus never came into direct contact with the CFTR channels recorded in the patch^78^. However, when we performed an experiment excising the patch of CFTR-containing membrane from the intracellular components, application of SMase directly to the patch caused no inhibition of CFTR^78^. If ceramide, phosphocholine, loss of sphingomyelin, or SMase directly inhibited CFTR, there would have been inhibition of CFTR in the excised patch. In contrast, if exposure to SMase induced a cellular signaling pathway that requires intracellular components, the response would be lost in excised patch experiments. Given that intracellular components in the cell-attached patch seemed to be important for inhibition, we hypothesized that bath-applied SMase interacted with lipids outside of the cell-attached patch, inducing a signaling mechanism that was transmitted to the channels within the patch.

Importantly, we also showed that inhibition of CFTR by SMase treatment occurs in bronchial epithelial cells collected from non-CF subject lungs. However, the mechanism in the *Xenopus* oocyte studies may not translate to the bronchial epithelial model, as the mechanism of inhibition of CFTR by SMase and its metabolites seems to be highly dependent on the cell type. For example, in T84 colonic epithelial cells, ceramide decreased apical chloride secretion by inhibiting basolateral cAMP-gated potassium channels via c-Jun N-terminal kinase (JNK)^79^. In Calu-3 cells, though, ceramide inhibited cAMP-dependent chloride currents via inhibition of CFTR itself^80^. The authors ruled out the involvement of protein phosphatase 2 A (PP2A), protein phosphatase 1 (PP1), and protein kinase C (PKC) using inhibitors and activators of these enzymes. No definite mechanism for inhibition of CFTR by ceramide in Calu-3 cells has been identified.

Ceramide, phosphocholine, and their derivatives activate many signaling cascades, but the specific enzymes involved in SMase-mediated inhibition of CFTR have not been determined in many cell types. The mechanism by which CFTR inhibition via SMase occurs in patient pulmonary epithelial cells is important to understand, especially since the clinically active therapeutic VX-770 cannot recover the channel activity, as seen in *Xenopus* oocytes^78^.

However, contrary to these studies finding inhibition of CFTR by ceramide, a recent study reports activation of CFTR by ceramide. The authors found that treatment of primary bronchial epithelial cells with Vasoactive Intestinal Peptide (triggering a cAMP and a calcium response) and Carbachol (triggering a calcium response) along with activating CFTR also generated ceramide platforms, within which CFTR accumulated^81^. Interestingly, the generation of these platforms appeared to be due to activation of acid-SMase on the surface of the cells. Treatment with amitriptyline to functionally inhibit acid-SMase (thus preventing an increase in ceramide) reduced CFTR activity, possibly due to decreased CFTR stability at the cell surface and therefor increased internalization. However, considering that the acid-SMase involved in ceramide platform generation was already located at the cell surface along with the understanding that amitriptyline acts by facilitating the degradation of lysosomal acid-SMase, these results appear hard to interpret. Given that ceramide is imbalanced in CF and has an effect on the activity of CFTR, the cycle of CFTR dysfunction, ceramide imbalance, and the effect of ceramide on CFTR needs to be understood to elucidate any feedback loops that are disrupted in CF or other inflammatory lung diseases.

### Lipid rafts affect CFTR activity

As mentioned previously, lipid rafts are microdomains in cell membranes that are enriched for cholesterol and sphingolipids. In murine tracheal epithelial cells, CFTR clusters into GM1-positive lipid rafts or platforms. Importantly, this clustering of CFTR and GM1 remains upon *P. aeruginosa* infection, one of the most common chronic infections in people with CF. Interestingly, *P. aeruginosa* also appears to cause the conversion of lipid rafts into ceramide platforms at the site of contact at the cell^32^.

In Calu-3 bronchial epithelial cells, removing cholesterol by treatment with cyclodextrin significantly decreased the amount of CFTR found in the detergent-resistant fraction, typically considered lipid rafts^82^. Aside from localization to these rafts, cholesterol is also important for CFTR motility at the cell surface, in that decreasing cholesterol increases motility, and increasing cholesterol decreases motility^34^. Considering that cholesterol is increased in CF cells, that CFTR’s binding partner NHERF1 requires cholesterol to maintain surface expression^58,59^, and that CFTR localizes to cholesterol-rich lipid rafts, it can be speculated that cholesterol upregulation is an attempt by the cell to increase CFTR’s stability at the plasma membrane. More research into this topic is necessary to fully understand cholesterol’s impact on CFTR activity and on the disease severity of CF and other inflammatory lung diseases.

## Conclusion

It is surprising that more work has not been done to characterize the effect of CFTR on cellular lipid compositions, and vice versa. Many other members of the ABC transporter family transport lipids, and mutations in many of these can cause human disease^83^. Furthermore, many other ion channels and membrane proteins display altered activity based on the surrounding lipid environment. Given that many lipids appear to be altered in CF lung epithelial cells, it is of vital importance to identify all of the lipid imbalances in CF and to study the activity of CFTR in this altered lipid state.

Regarding lipid imbalances in CF, experiments should be conducted to identify global lipid imbalances in the cell membranes of airway epithelial cells from people living with CF as compared with controls, and to determine the mechanisms for these imbalances. These experiments should take into consideration the class of CFTR mutation, so as to parse apart the effects of loss of CFTR’s ion channel function from a loss of proper CFTR trafficking. Furthermore, these experiments should determine the effects these lipid imbalances have on the health outcomes of people living with CF, and whether any methods of correction of the imbalance could positively impact the health of people living with CF. These experiments should also determine if the FDA-approved CFTR modulators rebalance the lipids. Even more specifically, though, experiments should be conducted that identify the specific lipid environment that CFTR experiences. Methods should be refined to purify CFTR in high-yield, high-quality batches that can be used to identify tightly-associated lipids.

In regards to lipids affecting CFTR activity, experiments at every level—from single-channel all the way up to a tissue or patient level—are needed to determine how the identified lipid imbalances are affecting the ability of CFTR to function. It is important that these experiments be validated in a relevant cellular model as well, with bronchial epithelial cells from people being the current gold standard.

Even if CFTR modulators are able to help CFTR function better, if CFTR is in a lipid environment that is not conducive to its function, those modulators are not going to work at their full potential. Improved treatment for CF is urgently needed. An enhanced understanding of CFTR-lipid interactions should not only provide a more accurate model of CF and other chronic lung diseases, but may aid in the identification of new drug targets, or lead to modulation of current therapies.
